# Impact of the COVID-19 pandemic on tobacco product consumption and behavioral patterns from a low-middle income country perspective: A qualitative study

**DOI:** 10.18332/tpc/201442

**Published:** 2025-03-12

**Authors:** Ana Paula Coelho Figureira Freire, Eric Foch, Bruna Aparecida Santos Medina, Juliana Souza Uzeloto, Marceli Rocha Leite, Bruna Spolador de Alencar Silva, Marina Politi Okoshi, Francis Lopes Pacagnelli

**Affiliations:** 1 Department of Health Sciences, Central Washington University, Ellensburg, United States; 2 Department of Physiotherapy, University of Western São Paulo, Presidente Prudente, Brazil; 3 Department of Physiotherapy, Educational Foundation of the Municipality of Assis, Assis, Brazil; 4 Department of Medicine, University of Western São Paulo, Guarujá, Brazil; 5 Department of Physiotherapy, São Paulo State University Júlio Mesquita Filho, Presidente Prudente, Brazil; 6 Department of Internal Medicine, Botucatu Medical School, Sao Paulo State University, Botucatu, Brazil; 7 Núcleo de Avaliação de Tecnologias em Saúde da Faculdade Medicina da Universidade do Oeste Paulista, São Paulo, Brazil

**Keywords:** smoking, smoking behaviors, smoking habits, pandemics, COVID-19 virus infection

## Abstract

**INTRODUCTION:**

Previous studies investigating socioeconomic status and tobacco consumption during the COVID-19 pandemic were survey-based. To extend knowledge beyond prevalence rates and trends of tobacco consumption, qualitative research is needed to identify individual's experiences. There is a critical gap within this context, particularly in low-middle income countries. The aim of the study was to perform a qualitative analysis on consumption patterns of tobacco users from a low-middle income country during the COVID-19 pandemic, and to identify factors influencing motivation to quit tobacco products during the pandemic and the perceptions of self-risk for complications of tobacco consumption and COVID-19.

**METHODS:**

A qualitative study was conducted in São Paulo, Brazil in September 2020. We used a focus group with semi-structured interviews. Participants were invited to answer questions about behavioral and consumption patterns of tobacco products during early stages of COVID-19 pandemic. Two investigators independently performed triangulation of content of the transcripts. Data were analyzed using inductive content analysis.

**RESULTS:**

Eighteen participants were evaluated (66.7% males) with mean age 34.1 ± 14.9 years. Many participants presented high levels (33.3%) of nicotine dependence. Thematic analysis of participants' narratives resulted in two themes: Theme 1: Behavioral and psychological factors impacting consumption; and Theme 2: Consumption patterns, dependence, and information. Open-coding process resulted on four codes: 1) Behavioral and lifestyle changes; 2) Psychological and motivational factors; 3) Consumption patterns and dependence; and 4) Information exposure and awareness. Nine categories were generated from the codes.

**CONCLUSIONS:**

Behavioral and consumption patterns varied significantly in tobacco users in Brazil during the early stages of the COVID-19 pandemic, ranging from increases to no changes. Individuals consuming tobacco products showed awareness about the harmful effects of smoking and COVID-19 complications.

## INTRODUCTION

Tobacco consumption is a serious public health challenge worldwide. Approximately 8 million people die annually due to tobacco related disease^1^. The COVID-19 pandemic has highlighted the potential health risks associated with tobacco consumption (both smoked and smokeless products) in the context of infectious respiratory diseases^2,3^. Tobacco consumption is associated with an increase of the risk of adverse events and fatal outcomes related to COVID-19^4-6^. Consumption patterns and motivation to quit during the pandemic differed significantly among countries of varying socioeconomic backgrounds^7-11^. In studies conducted in high-income European countries, increased consumption of tobacco product varied from 18.9% in the Netherlands^7^ to 45.2% in Poland^11^. Data from the US showed a 30.3% increase^9^, while in Brazil, a low-middle-income country, 44.2% of smokers reported an increase in consumption^8^. Middle-income countries are projected to have approximately a 20% higher prevalence of tobacco users compared to high-income countries^12,13^. Socioeconomic and healthcare disparities between countries might impact health behaviors. To gain a better understanding of how COVID-19 influenced tobacco consumption and health perceptions between tobacco and the coronavirus, insights are need from a low-middle income country demographic such as Brazil^14^.

Brazil was confronted with higher COVID-19-related mortality compared to other parts of Latin America and by May 2020, ranked third in the world in terms of total COVID-19 cases and deaths^15^. In June 2020, the country was second in viral infections and fatalities. Pandemic unpreparedness, a fragile healthcare system, and poor governmental support facilitated the spread of the virus throughout the country^15^.

Previous studies investigating socioeconomic status and tobacco consumption during the COVID-19 pandemic were survey-based^7-11,16^. This approach, although valuable, may not have captured the underlying factors related to tobacco consumption changes. To extend our knowledge beyond prevalence rates and general trends of tobacco consumption, qualitative research is warranted to study individual’s experiences and reflections^17,18^. Few studies qualitatively examined the effects of the COVID-19 pandemic on tobacco consumption, as well as the motivation and ability to quit smoking^19-21^. The studies that have conducted qualitative research were all performed in high-income countries and evaluated young adults^21^, elderly individuals^19^, and patients after hospitalization^20^. There is a critical gap in understanding the consumption behaviors of tobacco users within the context of the COVID-19 pandemic, particularly in low-middle income countries like Brazil.

Therefore, the purpose of the study was to perform a qualitative analysis on consumption patterns of tobacco users in Brazil, a low-middle income country, during the COVID-19 pandemic. The secondary purpose was to identify factors influencing the motivation to quit tobacco products during the COVID-19 pandemic and the perceptions of self-risk of complications from tobacco consumption and COVID-19.

## METHODS

### Study design

This was a qualitative study with data collection performed completely online in September 2020. The study followed the recommendations of the Consolidated Criteria for Reporting Qualitative Research (COREQ)^[Bibr CIT0022]22^. The study was conducted in São Paulo, Brazil, and participants completed an initial evaluation via an online survey, using the Google Forms platform^8^. Next, participants were invited to participate in focus groups via video conference. The focus groups included guided questions about behavioral and consumption patterns of tobacco products during the early stages of the COVID-19 pandemic (September 2020). Smartphones and computers, as well as training, were offered to individuals with difficulty accessing the video conferencing platform.

Participants were recruited from a sub-sample of a survey study conducted nationally in Brazil^8^. Recruitment strategies and sample size are described elsewhere^8^. Ninety-two individuals that participated previously^8^ were eligible and invited to participate. The sample for the current study was determined based on the available participants from the prior study who met the inclusion criteria. The sample size is consistent with previous studies utilizing similar methods21,23.

Inclusion criteria were individuals of both sexes who currently consumed any tobacco product frequently (at least 3 times a week) for at least one year, including regular cigarettes, cigars, rope tobacco, straw cigarettes (handmade cigarettes rolled using dried plant materials), tobacco pipes, waterpipe (hookah), and smokeless tobacco products (e.g. chewing tobacco). Both single and dual-product users were included. We included individuals aged >18 years with cognitive capacity to speak Portuguese and able to respond to study assessments. We excluded individuals that exclusively used electronic cigarettes, have been diagnosed with a severe psychiatric disorder (e.g. schizophrenia), or currently use marijuana, cocaine, or other narcotics.

This study was approved by the Institutional Research Ethics Committee of the University of Western São Paulo (Approval number: CAAE 30652220.2.0000.5515). Participants were informed of the study’s procedures and signed an informed consent form.

### Initial evaluation

For the current study, data regarding age, sex, body mass index (BMI, kg/m^2^), education level, and presence of comorbidities were included. Additionally, type of tobacco products consumed were included, and if necessary, individuals could select more than one type of product. The Fagerström test for nicotine dependence score was also assessed, this test is a screening instrument for physical nicotine dependence^24^.

### Focus groups

Focus groups were held virtually, due to COVID-19 restrictions, to collect qualitative data through an online meeting platform. Two focus groups were needed with the maximum number of participants being 12 per group. Twelve or less participants per group allows for optimal levels of engagement^17,22^.

Focus groups were conducted based on a semi-structured interview using five open-ended questions as a guide (Supplementary file). The interview was conducted and moderated by the principal investigator (APCFF) who has previous experience in qualitative studies. A second investigator (BASM) participated as a facilitator on the focus groups to take notes and register unexpected events. The open-ended questions were initially developed by the research team. Next, the questions received the input of an external expert and were tested in a pilot study and did not undergo any changes.

Participants were informed that the interview would be conducted by the principal investigator and facilitator, and given information on the researchers’ backgrounds. With the consent of the participants, interviews were audio and video recorded and later their de-identified data were transcribed verbatim. After each focus group interview was completed, the moderator and facilitator debriefed and shared their field notes. Focus groups were conducted to the point of saturation – the addition of participants did not result in the generation of new themes^23^.

### Data analysis

For the qualitative analysis, two investigators (BASM and JSU) independently performed the triangulation of the content of the transcripts^18,25^. Data were analyzed using an inductive content analysis, a systematic model to describe phenomena and refine words in codes related to the content^17,18^. Initially, researchers screened the transcripts for unit of analysis to identify common themes. Next, to organize the qualitative data, line-by-line open coding was performed. To refine the results, researchers formulated a general description of each topic by generating categories. Each category was named using content-characteristic words. The researchers debated the differences in the results to reach a consensus. Categorization was finalized through discussion and agreement between the two researchers^18^. Descriptive statistics were used to analyze participant characteristics, including mean and standard deviation (SD) for continuous variables and frequencies and percentages for categorical variables.

### Validity

The investigators shared the categorization of the transcripts with the focus groups participants to verify that the analysis clearly reflected the discussions of the focus groups. Participants that provided feedback (n=3) indicated that the results reflected the content of the focus groups discussions and did not make any changes to the categorization^17^. For transferability, the researcher established triangulation of the data and theoretical saturation^23,25^. Furthermore, the results were shared with two individuals who did not participate in the study, but satisfied the inclusion criteria, to review whether the findings aligned with their experiences^17^.

## RESULTS

Of the 92 individuals recruited from a previous study^8^, 57 either did not reply to the three invitations or declined to participate further. Seventeen participants did not attend the scheduled meeting for the focus group, despite initially accepting. The final sample included 18 participants (Table 1). The focus groups were carried out on two separate days, 14 days apart (16 and 30 September 2020). The same interview guide was used on both days. Seven individuals participated in the first focus group (36-minute recording). Eleven individuals participated in the second focus group (42-minute recording). The screening for unit of analysis resulted in two main themes:

**Table 1:** Participant demographic characteristics and tobacco use, São Paulo, Brazil, 2020 (N=18)

*Characteristics*	*Mean*	*SD*
**Age** (years)	34.05	14.91
**BMI** (kg/m^2^)	27.03	5.76
**Tobacco consumption** (years)	17.05	14.93
	***n***	***%***
**Sex**		
Female	6	33.33
Male	12	66.67
**Education level**		
Primary	1	5.56
Secondary	6	33.33
Tertiary	11	61.11
**Previous diagnoses**		
Psychological illness	8	44.44
Cardiovascular disease	4	22.22
Metabolic disease	1	5.55
Respiratory disease	1	5.55
**Fagerström classification**		
Very low	5	27.78
Low	2	11.11
Moderate	3	16.67
High	6	33.33
Very high	2	11.11
Tobacco product use*		
Regular cigarette	12	66.67
Straw cigarette	4	22.22
Electronic cigarette	1	5.56
Waterpipe	2	11.11
Cigar	1	5.56
Smokeless products	4	22.22

BMI: body mass index.

* Tobacco product use includes consumption of more than one type of product.

- Theme 1: Behavioral and psychological factors impacting consumption
- Theme 2: Consumption patterns, dependence, and information

**Figure 1:**
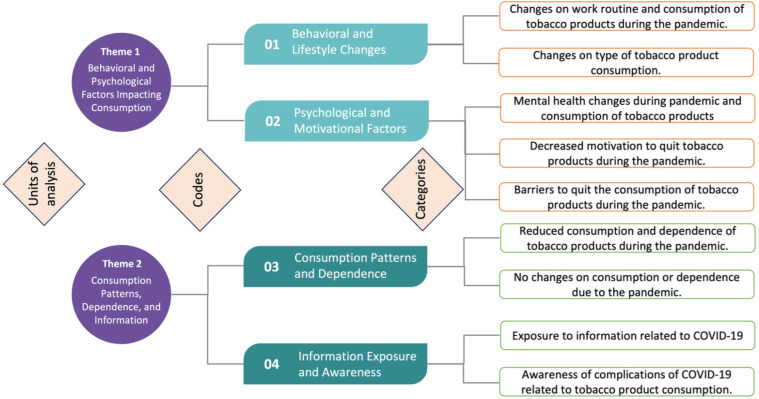
Coding tree: themes, codes, and categories

On the next stage of content analysis, researchers proceeded to an open-coding process that resulted in four main codes: 1) Behavioral and lifestyle changes, 2) Psychological and motivational factors, 3) Consumption patterns and dependence, and 4) Information exposure and awareness. Category consolidation refined the results of each code. Nine categories were generated (Figure 1).

### Theme 1: Behavioral and psychological factors impacting consumption

The pandemic affected both the type and amount of tobacco products used. Changes in workplace environment (office setting vs work from home) altered daily patterns and schedules facilitating the reported increased tobacco consumption. Conversely, some participants shifted their consumption patterns by substituting traditional cigarettes for other tobacco products such as hookah or pipe smoking, which they associated with more leisurely at-home habits rather than habitual smoking during work hours. Participants also reported that the pandemic also heightened psychological distress, anxiety, and fear, which participants identified as triggers for increased tobacco use. Furthermore, a decreased motivation to quit tobacco products was identified. Participants offered insight into the negative psychological factors experienced during the pandemic that led to antipathy towards quitting tobacco products (Table 2).

**Table 2:** Theme 1: Behavioral and psychological factors impacting consumption, São Paulo, Brazil, 2020 (N=18)

*Code*	*Category*	*Quotes*
**Behavioral and lifestyle changes**	Changes in work routine and consumption of tobacco products during the pandemic	*‘Look, I believe it increased [consumption] a little bit. Because of the freedom I have to smoke whenever I want. Before, working at the office, I had to use an elevator, face a huge line, and go down 10 floors to smoke. Now, nothing stops me from continuing to work [at home] and smoke at the same time. So yes, I believe my consumption increased.’* (Participant 1.1)
	Changes in the type of tobacco product consumed	*‘My consumption, I feel like it hasn’t increased. I feel like it has migrated between products. I smoked cigarettes and straw cigarettes, rope cigarettes and, now that I’m staying more at home, consumption has shifted from cigarettes to hookah and pipe. But I don’t feel like it’s increased, just changed the product.’* (Participant 5.2)
**Psychological and motivational factors**	Mental health changes during COVID-19 pandemic and consumption of tobacco products	*‘I think in my case it’s the anxiety. Anxiety and fear. Cigarettes are a refuge for this [the pandemic], for a little control over anxiety. With this pandemic, anxiety has increased, you become more nervous, you don’t know what will happen. You have a child, a mother, a father, you are afraid of someone catching the disease.’* (Participant 6.1)
	Decreased motivation to quit consumption of tobacco products during the pandemic	*‘I think the big reason to quit smoking [before the pandemic] was “I smell bad”, “I’m unpleasant”, or “I am disturbing someone”. Now, I don’t have that reason to motivate me anymore [working remotely], I don’t see any light at the end of the tunnel. So, I like to smoke. I’m on the computer, I’m smoking. I’ve eaten something, I’m smoking. Nothing stops me from smoking anymore, there’s so much bad stuff happening that I’m going to smoke, no doubt about it.’* (Participant 3.2)
	Barriers to quit the consumption of tobacco products during the pandemic	*‘You become more anxious, more nervous, afraid of someone in your family catching this disease and you end up compensating with cigarettes. So, I think that during this period, I believe it will be more difficult [to quit], I think that when this pandemic pass, I believe it will become easier to try to quit.’* (Participant 1.1)

### Theme 2: Consumption patterns, dependence, and information

This theme generated a better understanding on how consumption patterns were affected. Changes in tobacco use varied among participants. While some reported an increase in consumption, others reduced or maintained their usage levels. Fear of COVID-19 complications emerged as a motivating factor for reduction or cessation. There were reports of reduction and even attempts to cessation during the pandemic. The awareness and access to information related to the pandemic, were also addressed. Regarding exposure to information, participants expressed difficulty in navigating conflicting reports about smoking and COVID-19 risks. Despite these uncertainties, many participants acknowledged the increased health risks associated with tobacco use and COVID-19 (Table 3).

**Table 3:** Theme 2: Consumption patterns, dependence, and information, São Paulo, Brazil, 2020 (N=18)

*Code*	*Category*	*Quotes*
**Consumption patterns and dependence**	Reduced consumption and dependence of tobacco products during the pandemic	*‘At the beginning of the pandemic I kept trying to stop [smoking] and couldn’t, I kept smoking. But since August, I stopped because I was afraid of dying, I was overweight and that sort of thing … I lost 10 kg. But it’s more of … it was the fear of dying.’* (Participant 7.2)
	No changes in consumption or dependence due to the pandemic	*‘I haven’t changed my smoking habit much. I smoked a pack a day and continue to smoke a pack a day. So, there wasn’t much change. I stopped working for a few days at the beginning of the pandemic, 15 days, but then I went back to work normally so my daily life remained normal, I continue to smoke the same amount of cigarettes.’* (Participant 6.1)
**Information exposure and awareness**	Exposure to information related to COVID-19	*‘Due to various news reports, we didn’t know what was true, what wasn’t. I even heard that people who smoked were less likely to get it, I never believed that either.’* (Participant 1.2)
	Awareness of complications of COVID-19 related to tobacco product consumption	*‘Assuming that this disease will attack the lungs, every smoker’s lungs are already aggravated lungs. So, if we do get a disease, our risk of dying is higher than that of a non-smoker, because we already have much lower lung and breathing capacity too, so I think the risk is very serious in this case.’* (Participant 1.1)

## DISCUSSION

The purpose of this qualitative study was to investigate the influence of the COVID-19 pandemic on tobacco consumption patterns, as well as the motivation to quit tobacco products from a low-middle income country perspective. Two main themes were identified: Theme 1: Behavioral and psychological factors impacting consumption, and Theme 2: Consumption patterns, dependence, and information.

Lifestyle changes during the pandemic, particularly work routine, affected the behavior around tobacco consumption patterns. Our sample consisted of mostly working young adults that reported the change to working from home and a lack of options to leave home for recreation (e.g. social engagements and exercising) were the primary reasons to increase tobacco product consumption. Previous qualitative studies conducted in the US also identified that a less structured schedule was one of the main reasons for increased smoking^26^.

Our findings are also in agreement with previous mixed-method studies that found social isolation can increase levels of stress and serve as a trigger for increased tobacco consumption^27^. Smoking and the use of smokeless products was a coping mechanism while in a stressed psychological state^28^. In low- and middle-income countries the distress can be even higher. The effects of the pandemic on the economic and social sectors in these countries resulted in many suffering a complete loss of earnings, since many are employed in the informal labor market^27^.

We also identified a category related to the influence of mental health changes during the pandemic and increased consumption of tobacco products. Many studies investigated the relationship between tobacco use and potential negative effects, anguish, or anxiety among individuals in situations of stress^29-31^. Theories have been provided to explain the reason individuals in stressful situations tend to smoke more. The evaluation of smokers with post-traumatic stress disorder^30^ and survivors of weather disasters^31^ showed that smoking was used as a self-medication tool to relieve feelings of depression or anxiety. Conversely, other studies suggest that smoking can cause mental health issues by altering an individual’s neurocircuitry. The hypersecretion of cortisol might cause smokers to become more vulnerable to environmental stressors^29^. During the COVID-19 pandemic, the continued uncertainties surrounding the disease transmission, apprehensions regarding the health of loved ones, and the containment measures have consistently emerged as themes related to stress in prior qualitative analyses^20,27^.

Our analysis also identified differences in how tobacco users altered their tobacco consumption habits differently during early stages of the pandemic. Some participants did not change their consumption patterns or even reduced it due to concerns about the combined health impacts of COVID-19 and smoking. The fear of complications was a motivational factor to decrease consumption, and several participants reported a good level of awareness on the risks of smoking and health outcomes related to COVID-19. Regardless of the changes in consumption patterns, it is important to highlight that encouraging cessation of tobacco products is essential. However, specific approaches might be needed in pandemic scenarios. During a pandemic, barriers to treatment access may arise, potentially compromising access to health services. The access could be limited due to social distancing policies. In addition, in-person support for tobacco users from healthcare workers can be restricted, thus, leading to challenges in both the initiation and continuation of cessation treatment.

Participants consuming tobacco products acknowledged the harmful effects of smoking on lungs and the possible complications of these effects related to COVID-19. In contrast, participants also reported that they felt overwhelmed with the high volume of information from media channels (e.g. associated press news reports, and social media). Some participants struggled to differentiate reliable information from fake news (e.g. smoking could be a protective factor for COVID-19). Fake news proliferation increased globally during the COVID-19 pandemic^32^. Therefore, health literacy and educational strategies are needed, particularly for individuals who have a higher tendency to engage in fake news dissemination including low income, younger, males, high internet/social media use, and low education^32^.

### Strengths and limitations

Our work adds to the literature by building on quantitative research to explain triggers or barriers to tobacco use during early stages of the COVID-19 pandemic. Additionally, most qualitative data available comes from high income countries, whereas our study analyzed the perceptions of a population that was overlooked in the literature (low-middle income country).

The study has some limitations. Individuals of low socioeconomic status usually do not reach web surveys. Therefore, our sample might not accurately reflect all segments of the target population. We offered, however, technology resources to facilitate participation. Alternative recruitment strategies in future studies are greatly needed to equitably evaluate vulnerable populations. Additionally, our findings are limited in their generalizability to high-income (or low-income) countries. Moreover, it is important to consider the rapidly evolving nature of the COVID-19 pandemic and related public health policies across time. As the pandemic progressed (after 2020), new variants of the virus, changing government regulations, and shifting societal behaviors may have altered tobacco consumption patterns and attitudes toward smoking cessation. Therefore, the findings from our study reflect the situation at a specific time point, and future studies would be needed to capture the long-term impacts of the pandemic and its effects on tobacco use behaviors. Finally, although consistent with qualitative research methods, our sample size is small even when compared to similar work. Therefore, future studies addressing these issues are needed to further advance this knowledge.

## CONCLUSIONS

Behavior around tobacco consumption patterns varied significantly during early stages of the COVID-19 pandemic. Participants reported a tobacco consumption pattern that was either reduced, increased, or remained unchanged. Regardless of any change in tobacco consumption, participants showed proper knowledge of the harmful effects of smoking and the possible complications related to COVID-19. Collectively, the importance of addressing psychosocial factors in tobacco control efforts cannot be underscored. Increased stress, anxiety, and lifestyle changes influence consumption behaviors and highlight the need for targeted mental health support and smoking cessation strategies during public health crises. Additionally, the spread of misinformation about tobacco use and COVID-19 highlights the important role of public health communication in ensuring access to accurate information, particularly in low-middle income country settings.

## Supplementary Material



## Data Availability

The data supporting this research are available from the authors on reasonable request.
