# Supplementary material for: Impact of the COVID-19 pandemic on tobacco product consumption and behavioral patterns from a low-middle income country perspective: A qualitative study
Source: Tob Prev Cessat. 2025 Mar 12;11:10.18332/tpc/201442. doi: 10.18332/tpc/201442 (PMC11898110; doi:10.18332/tpc/201442)
Supplement: Supplementary file 1 [file TPC-11-16-s1.pdf]

Supplementary material. Semi-structured interview guide. Qualitative study. São Paulo, Brazil, 2020.

	**Questions**
1	Did you change your consumption of tobacco products since the beginning of the COVID-19 pandemic? If changes were observed, please explain possible reasons.
2	Do you feel more motivated to quit the consumption of tobacco products during the COVID-19 pandemic? Explain possible reasons for your answer.
3	Do you believe that consumers of tobacco products are more likely to develop complications or more severe forms of COVID-19 when compared to non-consumers?
4	Do you feel more dependent on tobacco products during the pandemic? If changes were observed, explain how and possible reasons.
5	Do you believe it would be harder or easier to quit the consumption of tobacco products during the COVID-19 pandemic? Explain possible reasons for your answer.

© 2025 Freire A.P.C.F. et al.
